# Berberine ameliorates mesenteric vascular dysfunction by modulating perivascular adipose tissue in diet-induced obese in rats

**DOI:** 10.1186/s12906-022-03667-1

**Published:** 2022-07-25

**Authors:** Man Wang, Xufang Geng, Kaipeng Li, Yawen Wang, Xiaofeng Duan, Congcong Hou, Lili Zhao, Huimin Zhou, Ding Zhao

**Affiliations:** 1 College of Pharmacy, Hebei Medical University, No. 361 Zhongshan East Road, Shijiazhuang, 050017 People’s Republic of China; 2 Hebei Academy of Agriculture and Forestry Sciences, Shijiazhuang, People’s Republic of China; 3 The First Hospital of Hebei Medical University, Shijiazhuang, People’s Republic of China

**Keywords:** Obese rats, Berberine, Mesentery, Perivascular adipose tissue, Metformin

## Abstract

**Background:**

Berberine (BBR) has been found to have antiobesity effects, and obesity can lead to adipose tissue degeneration. As a special adipose tissue, perivascular adipose tissue (PVAT) is closely related to vascular function and affects vasoconstriction and relaxation. What happens to PVAT in the early stages of diet-induced obesity and how BBR affects vascular function is the focus of our experimental study.

**Methods:**

Sprague–Dawley rats were fed a high-fat diet (fat 34% kcal) for 4 weeks to simulate early obesity. Obese rats were treated with BBR (200 mg/kg) or metformin (MET, 100 mg/kg) by gavage for 2 weeks. The mesenteric arterioles were studied by atomic force microscopy (AFM). The force vs. time curves were observed and analysed to indicate vascular function. Nitric oxide (NO) and noradrenaline (NA) release was quantified using an organ bath with fluorescence assays and ELISA, respectively. Network pharmacology was used to analyse the overlapping targets related to BBR and obesity-related diseases, and the expression of NOS in mesenteric PVAT was further analysed with immunohistochemistry and real-time PCR. The serum inflammatory factor levels were tested.

**Results:**

BBR significantly reduced the levels of blood glucose, blood lipids and inflammatory factors in serum. It also effectively improved abnormal mesenteric vasoconstriction and relaxation in obese rats. There was no significant change in mesenteric vascular structure, but NO production and eNOS expression were significantly increased in mesenteric PVAT (*P* < 0.01), and NA was decreased (*P* < 0.05) in obese rats. All these changes in the mesenteric arterioles and PVAT of obese rats were reversed by treatment with BBR and MET.

**Conclusions:**

In diet-induced obesity in rats, the function of vasoconstriction and relaxation in mesenteric arterioles is altered, NO is increased, and NA is decreased in mesenteric PVAT. All these changes were reversed by BBR, suggesting a novel effect of BBR in ameliorating mesenteric vascular dysfunction by regulating PVAT.

## Background

In recent years, an increasing prevalence of obesity has been observed in many countries, and one-third of the worldwide population is described as obese or overweight [1]. Obesity is a risk factor for developing many disorders, such as diabetes mellitus, hypertension, and cardiovascular events [2]. Moreover, obesity increases oxidative stress, induces an inflammatory state, and induces hypoxia, all of which contribute to adipose tissue dysfunction. Perivascular adipose tissue (PVAT) is an additional special type of adipose tissue that surrounds blood vessels, and it has been demonstrated to regulate vascular tone and wall remodelling [3]. The inhibitory regulation of the contractile responses by PVAT was first reported in normal rat aortas by Soltis and Cassis [4]. In addition, the functional importance of the mesentery was first proposed in 2016 [5], and in our early studies, the mesenteric PVAT was the sensitive part of the lesion in early diabetic rats [6]. Although there have been reports about mesenteric PVAT increasing vasorelaxation in obese rats [7] (feeding a high-fat diet for 8 weeks), reports from the perspective of early prevention are lacking. What are the changes in mesenteric PVAT in early obesity? Does it affect blood vessel function?

Berberine (BBR) is a natural compound found in numerous herb plants, including *Coptis chinensis* and *Berberis vulgaris*, and it is an alkaline antibacterial drug that is used to treat diarrhoea [8] and dysentery [9]. BBR has received much attention in recent decades because of its significant antidiabetic [10] and antiobesity [11] effects that act through multiple targets and pathways, especially anti-inflammatory pathways and protective pathways against oxidative stress [12]. The effect of BBR on vascular function was mainly focused on endothelial-derived NO instead of PVAT. In both cultured endothelial cells and aortas isolated from rats, BBR enhanced the phosphorylation of eNOS, leading to increased production of NO [13]. We have reported that BBR has protective effects against HUVEC damage induced by vanadium compounds. This process is not mediated by eNOS but is related to reduce intracellular ROS [14]. We also reported that in the early stages of STZ-induced diabetes in rats, neural control of mesenteric and iliac vasomotor tone are altered differently, and the diminished nitrergic nerve on the superior mesenteric artery and enhanced adrenergic nerve on the iliac artery both contributed to increased vasoconstrictor responses. All these changes in diabetic rats were reversed by BBR [15]. Many studies have shown that MET and BBR share many aspects in their actions and mechanisms [16–18]. However, MET, but not BBR, was widely used to treatment of T2DM and obesity. So in this study, we compared the effects of the two drugs, trying to provide a research basis for the clinical expanding application of BBR.

Thus, we hypothesized that BBR improves the obesity-induced mesenteric vascular dysfunction by regulating the release of paracrine vasorelaxing/vasocontracting factors in PVAT and compared the effects of BBR with MET. In the present study, we first investigated whether changes in mesenteric vascular function occur early in the development of high-fat diet (HFD)-induced obesity in rats and the possible mechanisms involved. We next investigated whether the effects of BBR on mesenteric vascular dysfunction are associated with PVAT. This study may provide new insights on the usage of BBR. Mesenteric arteriole function was studied by atomic force microscopy (AFM). The NO and NA released by PVAT in the mesenteric vasculature were quantified to indicate the changes in PVAT. Network pharmacology was used to analyse the overlapping targets related to BBR and obesity-related diseases, and the expression of NOS in mesenteric PVAT was further analysed with immunohistochemistry and real-time PCR.

## Methods

### Establishment of the obesity model and treatment strategy

Forty six-week-old male Sprague–Dawley rats (160–180 g) were purchased from the Experimental Animal Center of Hebei Medical University (China). They were housed in groups of four and given three days to acclimate to the housing facility. The environmental conditions were a temperature of 21 °C ± 2 °C, humidity of 55% ± 10% and a 12:12 light: dark cycle with lights on at 07:00 and off at 19:00. Animals were housed in 600 × 400 × 300 mm cages and given access to rat maintenance food and water ad libitum. During housing, animals were monitored twice daily for health status. No adverse events were observed. The rats were divided into a control group (CON), an obesity model group (OBE), and BBR and MET administration groups (OBE + BBR, OBE + MET) by simple random sampling. Table 1 shows animal experimental design, the rats in the control group were fed a normal diet (fat 13% kcal); the rats in the other three groups were fed a HFD (fat 34% kcal, Beijing Botai Hongda Biotechnology Co., Ltd, China). After two weeks, the rats fed a HFD with an obesity degree over 20% were determined to be successful in the obesity model [19] (control group *n = *8, obesity model group *n = *8, BBR administration group *n = *8, and MET administration group *n = *8). Then, the rats were concurrently treated with the corresponding agents intragastrically as follows for two weeks: the control and obese rats received equivalent amounts of saline solution, the BBR administration group was administered 200 mg/kg BBR (25 mg/mL, Sichuan Xieli Pharmaceutical Co., Ltd), and the MET administration group was administered 100 mg/kg MET (12.5 mg/mL, Shouguang Fukang Pharmaceutical Co., Ltd). The dosages of BBR and MET were adopted based on reports from our laboratory and other previous studies [15, 20, 21]. All animals were handled in accordance with our institutional guidelines for animal care, as well as with the National Institutes of Health guidelines regarding the care and use of animals for experimental procedures. The present study was approved by the Hebei Medical University Ethics Committee for Animals (SYXK (Ji) 2020–002). The study was conducted in compliance with the ARRIVE guidelines. After the experimental period, the rats were anaesthetized with an anaesthesia machine at an induction concentration of 3–4% isoflurane. Then, the abdominal cavity was opened at a maintenance concentration of 2–2.5%, and blood was collected from the abdominal aorta. After the blood was collected, the animals were euthanized by anaesthesia, and the mesenteric secondary vessels were collected. We identify the secondary mesenteric vessels: firstly, we determine the mesenteric aorta, the first branch of the aorta is the mesenteric primary vessels, and the small branches of the primary vessels are the mesenteric secondary vessels.

**Table 1:** Animal experimental design

Group	1–2 weeks	3–4 weeks
CON	normal diet	normal diet
OBE	high-fat diet	high-fat diet
OBE + BBR	high-fat diet	high-fat diet + BBR
OBE + MET	high-fat diet	high-fat diet + MET

### Serum analysis

Rats were anaesthetized, the abdominal cavity was opened, and blood was collected from the abdominal aorta. After incubating at room temperature for 30 min, the blood was centrifuged at 4 °C and 2000 r/min for 10 min. Then, the serum was collected and stored at -40 °C. The levels of blood glucose (GLU), total cholesterol (TC) and triglycerides (TGs) in serum were detected by an automatic biochemical analyser (Hitachi Hight-tech, Japan). Commercial enzyme-linked immunosorbent assay (ELISA) was used to evaluate serum interleukin-6 levels (Rat IL-6 EK306-96 T, MULTI SCIENCES, China), interleukin-1beta (Rat IL-1β EK301BV2-96 T, MULTI SCIENCES, China), and tumour necrosis factor-alpha (Rat TNF alpha Uncoated ELISA 88–7340, Thermo Fisher Scientific, USA) levels.

### Mechanical parameters by AFM

#### The sensitivity measure of AFM [22]

The probe (Tipless Probe, Mikro Masch, USA) was installed on the AFM (Agilent Technologies, USA). In contact mode, after setting the parameters, the probe sensitivity (nm/V) was obtained by measuring the force curve in the culture dish base. Concurrently, the force constants (N/m) of each probe were recorded.

#### Force vs. time curve acquisition

When the rats were anaesthetized with isoflurane, the abdominal cavity of the rats in each group was dissected. Then, the mesentery of the small intestine was pulled out and fixed on the sample stage. Then, the mesenteric vessel was localized by a charge-coupled device camera (CCD). In contact mode, the probe was driven to approach and contact the vessel. Vasomotion will affect the probe’s floating, and AFM will record the real-time force vs. time curves. During the experiment, the mesentery was infused with saline regularly to maintain the moisture levels of the tissue.

### Analysis of BBR by network pharmacology

With BBR as the key word, 87 BBR-related targets were obtained from STITCH (http://stitch.embl.de/, ver. 5.0). We retrieved related targets for six representative obesity-related diseases in GeneCards (https://www.genecards.org/) and screened the highly correlated protein targets (relevance score ≥ five times the median). A total of 812 obesity-related targets, 412 diabetes mellitus type 2-related targets, 546 fatty liver-related targets, 1118 hypertension-related targets, 208 hypercholesterolemia-related targets, and 227 hyperglycaemia-related targets were screened. After removing duplicate values, 1928 targets related to obesity-related diseases were obtained. Targets related to BBR and obesity-related diseases were obtained using network pharmacology analysis. Fifty-five duplicate targets were screened. Cytoscape 3.8.2 software (https://cytoscape.org/) was used to build the network.

### Determination of NO and NA production

For the electrical field stimulation (EFS) experiment, the vessels were mounted between two platinum electrodes placed 0.5 cm apart and connected to a current stimulator (S48 Stimulator; Grass Instruments, USA). The tension across the vessels was recorded before and after EFS using a physiological recorder (PL3508 Power Lab biological signal acquisition system; AD Instruments).

The secondary mesenteric vessel segments (without stripping the surrounding adipose tissue) were incubated with a fluorescent probe (4, 5-diaminofluorescein, DAF-2; 2 μmol·L^−1^) for 45 min in an organ bath. Then, the medium was collected to measure basal NO release. Subsequently, EFS was applied (frequency: 1, 2, 4, 8, and 16 Hz; cumulative stimulation; 20 s stimuli applied at 1-min intervals), and the medium was collected once more to measure NO and NA release. The fluorescence of the medium was measured using the spectrofluorometer of a microplate reader (Molecular Devices, Spectra Max i3, USA) with the excitation wavelength set at 492 nm and emission wavelength at 515 nm [23]. NA release was assessed using an NA ELISA kit (Human Noradrenaline ELISA Kit CSB-E07022r, Arigo Laboratories, China), followed by measurement of absorbance using a microplate reader.

The fresh secondary mesenteric vessels were stabilized in Krebs–Henseleit (K-H) (30 min, 37 °C), stained with DAF-2 (10^–5^ M), washed 2 × 15 min in K-H, and then fixed in 4% paraformaldehyde. Finally, the tissue was observed under a fluorescence microscope (Leica Microsystems, DMI4000B, Germany) [7].

### Histomorphology and immunohistomorphology

The 4% paraformaldehyde-fixed and paraffin-embedded tissue (secondary mesenteric vessels without stripping the surrounding adipose tissue) sections were cut into 5 μm-thick sections and stained with haematoxylin and eosin (H&E), Masson’s trichrome and immunohistochemical staining with a standard procedure. Rabbit anti-rat eNOS (1:100; BD Transduction Laboratories, USA) and rabbit anti-rat iNOS (1:100; Arigo Biolaboratories, China) were used for immunohistochemistry.

### Real-time PCR assays for eNOS

TRIzol reagent (Servicebio, China) was used to extract RNA from PVAT. Total RNA was retrotranscribed to synthesize cDNA with the help of a Verso DNA Synthesis Kit (Thermo Fisher Scientific, USA). The primers used for rat eNOS were 5’ GGTATTTGATGCTCGGGACTGC 3’ and 5’ GTGATGGCTGAACGAAGATTGC 3’, and GAPDH was used as a reference gene. The 2^−ΔΔCt^ method was used to calculate the relative fold change [24].

### Statistics

Analyses of the imaging data were carried out using SPSS 21.0 statistical software (International Business Machines Corporation, USA). The data were tested for normality firstly, and the differences among multiple groups were analysed using ANOVA (one-way analysis of variance), followed by the LSD test. For all tests, the level of significance was set at *P* < 0.05.

## Results

### Abnormal blood glucose and blood lipid levels in early obesity is accompanied by inflammation in rats

**Fig. 1:**
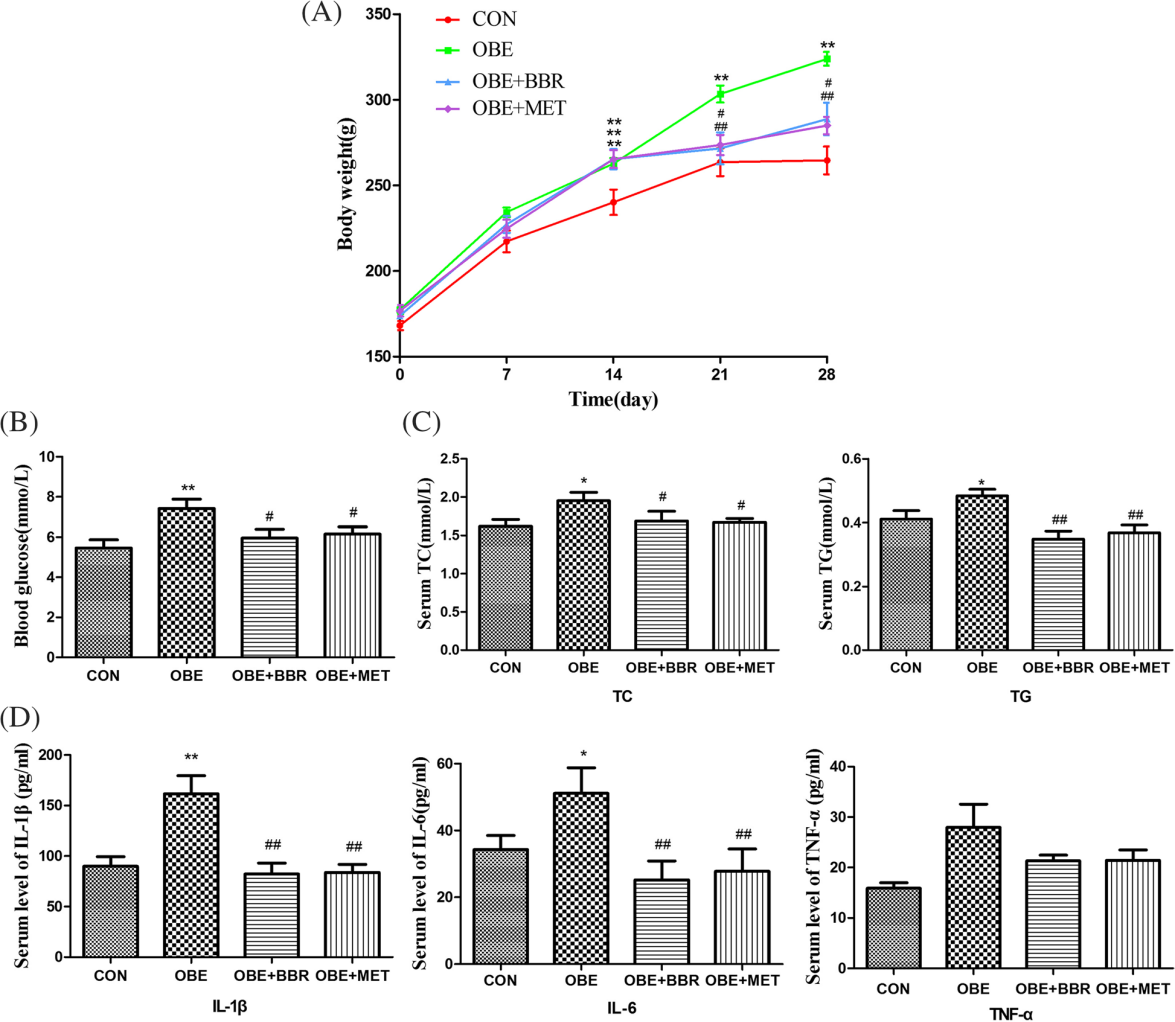
Abnormal blood glucose and blood lipid levels in early obesity in rats is accompanied by inflammation. **A** Body weight of rats. **B** Serum levels of blood glucose. **C** Serum levels of blood lipids. **D** The levels of IL-6, IL-1β and TNF-α in serum. Data are expressed as the mean ± S.E.M. (*n = *8). ^*^P < 0.05, ^**^P < 0.01 vs. control rats; ^#^*P* < 0.05, ^##^*P* < 0.01 vs. obese rats

The rats were fed a high-fat diet for four weeks, and their body weight was monitored. Serum was obtained to detect the levels of blood sugar, blood lipids and inflammatory factors. Obese rats demonstrated a significant increase in body weight, blood glucose and blood lipid levels, and inflammation (Fig. 1). However, the administration of BBR or MET significantly inhibited weight gain, significantly improved blood sugar and blood lipid abnormalities, and simultaneously corrected inflammation (Fig. 1). Our experimental results show that the above indicators were changed in early obesity in rats, and the interventional administration of berberine and MET improved these effects.

### The atomic force microscope (AFM) micromechanics of microvessels as an indication of vascular function

**Fig. 2:**
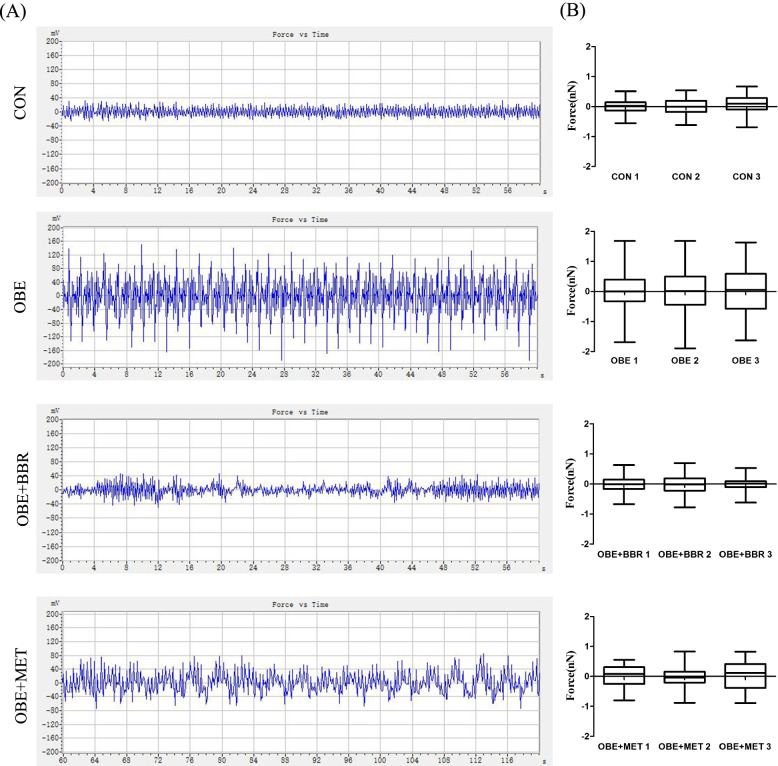
Atomic force microscope (AFM) micromechanics of micro vessels as an indicator of vascular function. **A** Force vs. time curve of secondary mesenteric vessels. **B** Box chart showing the distributions of contraction force. Data are expressed as the mean ± S.E.M. (*n = *3)

When the probe gradually approaches the surface of the sample, force is generated between the atoms on the tip of the probe and the atoms on the surface of the sample. Force (nN) = force constant (N/m) × degree of vasodilation (nm). That is, the greater the degree of vasodilation is, the greater the force on the probe. This is reflected in Fig. 2A by the greater the curve fluctuation. Therefore, these results indicate that the degree of vasodilation in obese rats is increased, and the vascular function is changed. Furthermore, both BBR and MET reversed the overvasodilatation (Fig. 2A). These data were mapped with GraphPad Prism software to directly present the data of each animal in each group (Fig. 2B).

### Vascular and mesenteric tissue morphology

**Fig. 3:**
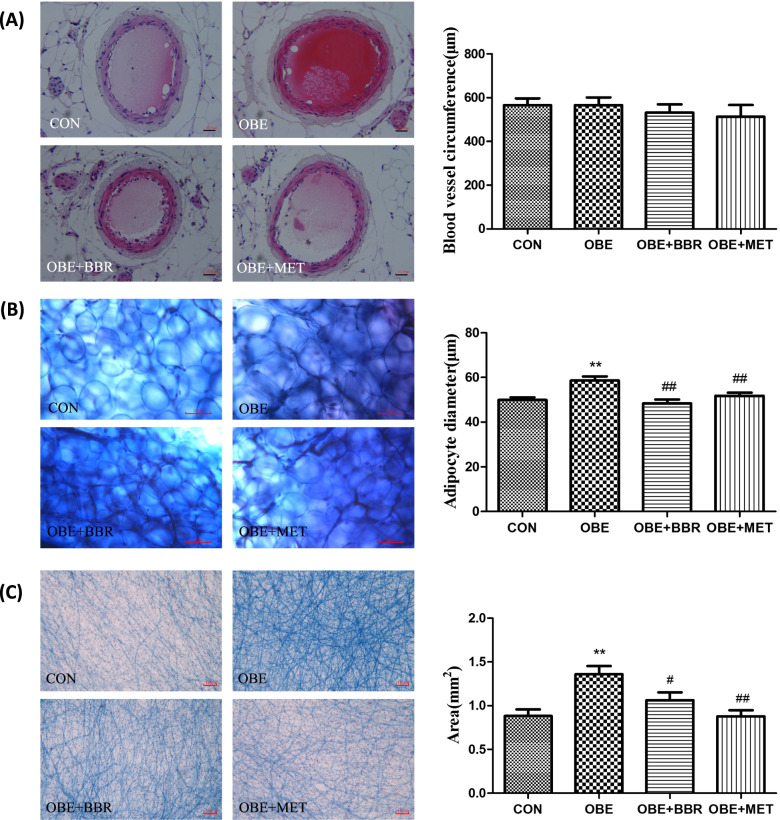
Mesentery and mesenteric vessel histomorphology. **A** HE staining of secondary mesenteric vessels (bars = 100 μm) and measurement of vessel circumference. **B** Masson staining of adipose tissue around mesenteric vessels (bars = 50 μm) and measurement of the adipocyte diameter. **C** Masson staining of mesentery in rats (bars = 100 μm) and measurement of the area of collagen fibres. Data are expressed as the mean ± S.E.M. (*n = *5). ^**^P < 0.01 vs. control rats; ^#^*P* < 0.05, ^##^*P* < 0.01 vs. obese rats

The secondary mesenteric blood vessels were stained with HE, and their circumference was measured. We found no significant changes in the morphology or circumference of the blood vessels (Fig. 3A). The mesenteric tissue was stained by Masson’s trichrome, and the collagen fibres are shown in blue. Compared with the control rats, the volume of adipocytes around the mesenteric vessels and the collagen fibres were significantly increased in the obese rats; BBR and MET reversed these increases (Fig. 3B, C). Image-Pro Plus software was used to measure and analyse the volume of adipocytes and the area of collagen fibres in each group. These results indicate that the tissue structure of the mesenteric blood vessels has not yet changed during early obesity in rats, but the fat tissue and fibres around the blood vessels have changed.

### Targets for vascular dysfunction in obesity-related diseases

**Fig. 4:**
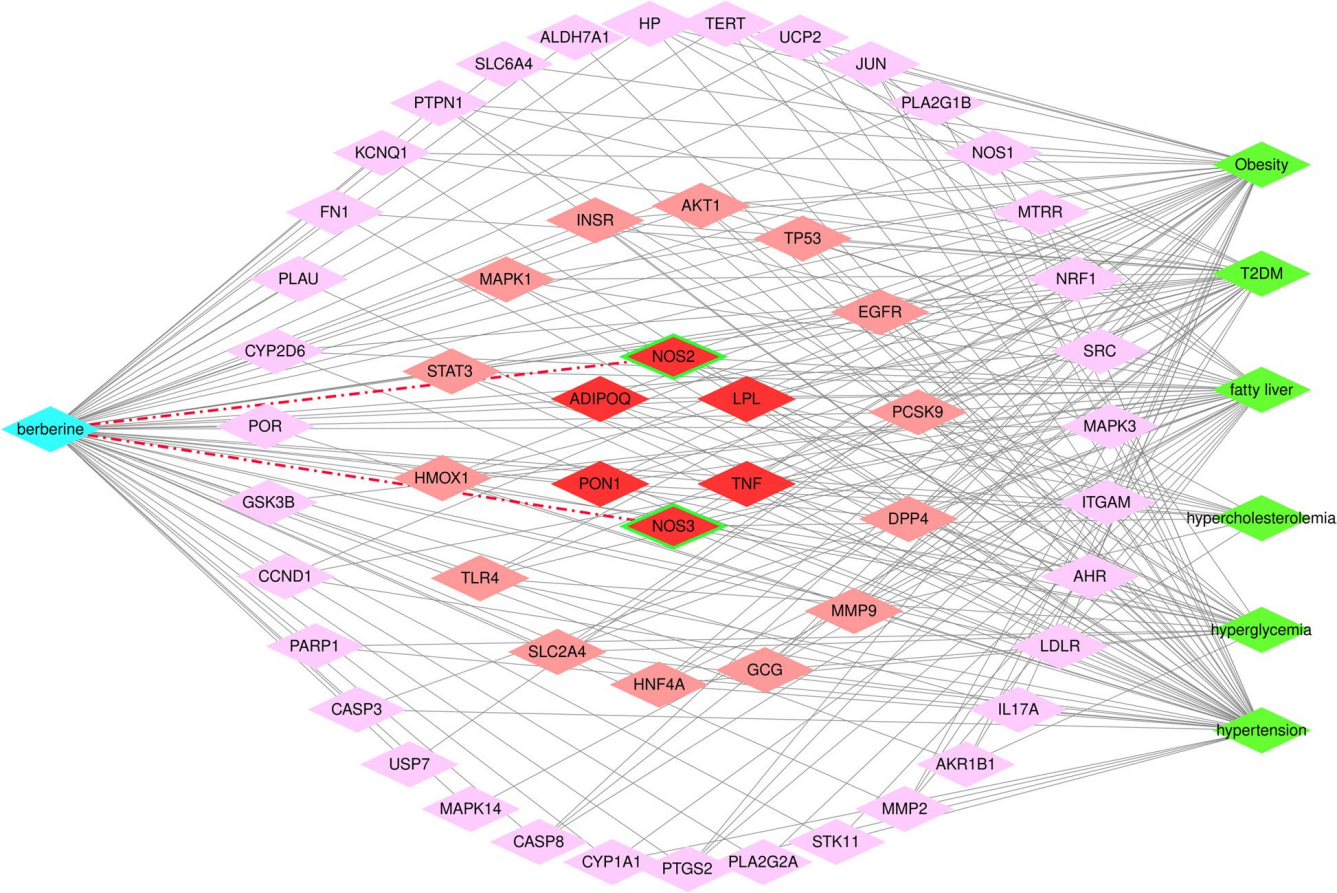
A network of berberine and obesity-related diseases predicted by potential protein targets. The blue nodes represent berberine, the green nodes represent the diseases, and the red nodes represent the protein targets. For protein targets, the deeper the red is, the stronger the correlation is. There were six highly correlated targets in total

Based on the overlapping target prediction, we tried to find a basis for the next step in the study of the targets of vascular dysfunction. As shown in Fig. 4, the BBR network of obesity-related diseases (6 diseases) predicted 55 common potential protein targets. The six diseases mentioned in network pharmacology are relatively common diseases that are closely related or even secondary to obesity. It is hoped that the analysis of common targets of obesity and closely related diseases will provide big data support for further research priorities. There are a total of six highly related goals; among them, NOS3 (eNOS) and NOS2 (iNOS) are related to vascular function. We will focus our experiments on the changes in vascular NO release and the expression of NOS.

### Changes in NO and NA release from secondary mesenteric blood vessels (including PVAT)

**Fig. 5:**
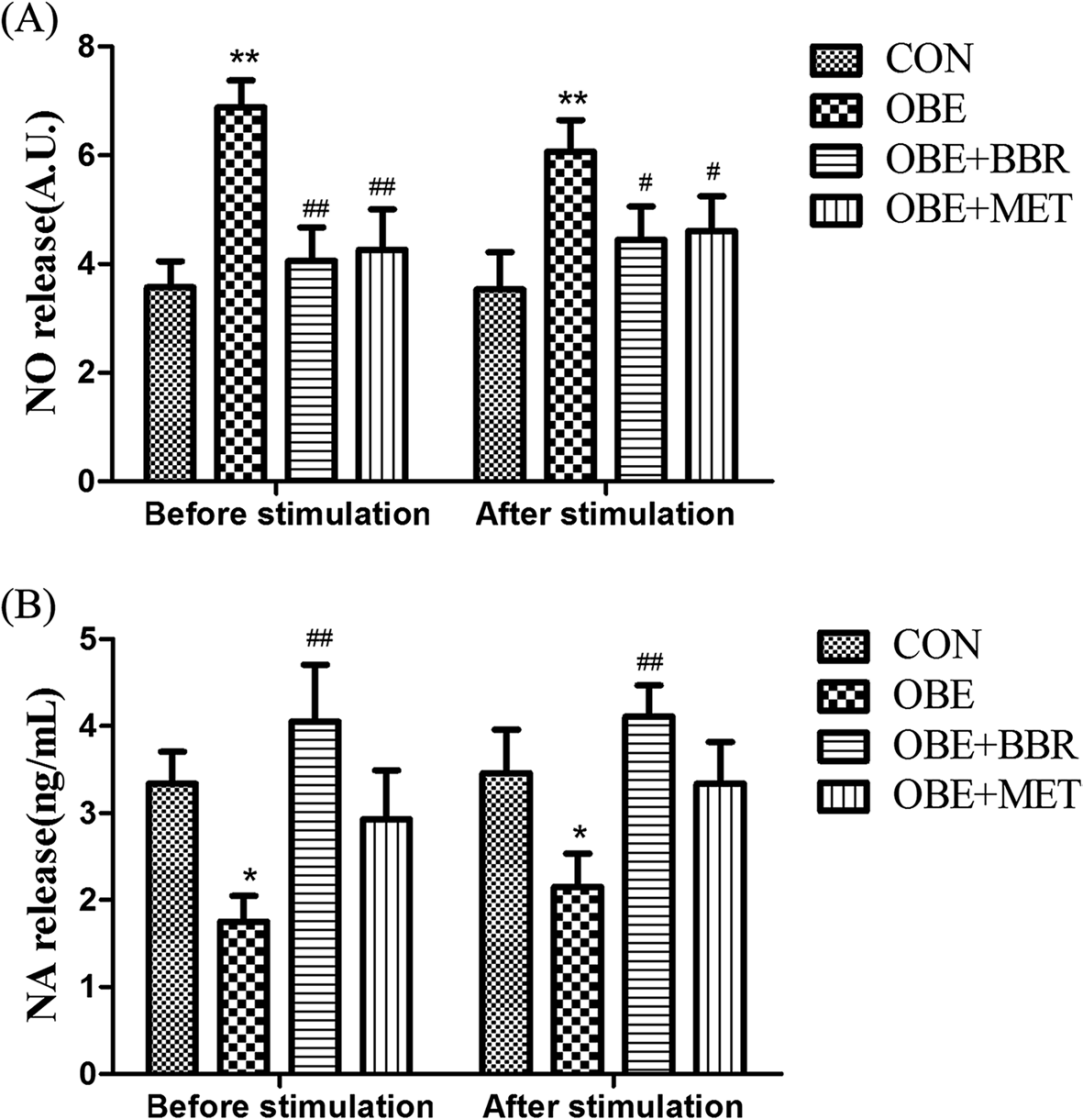
Changes in NO and NA release from secondary mesenteric vessels. **A** NO release from secondary mesenteric vessels. **B** NA release from secondary mesenteric vessels. Data are expressed as the mean ± S.E.M. (*n = *8). ^*^P < 0.05, ^**^P < 0.01 vs. control rats; ^#^*P* < 0.05, ^##^*P* < 0.01 vs. obese rats

**Fig. 6:**
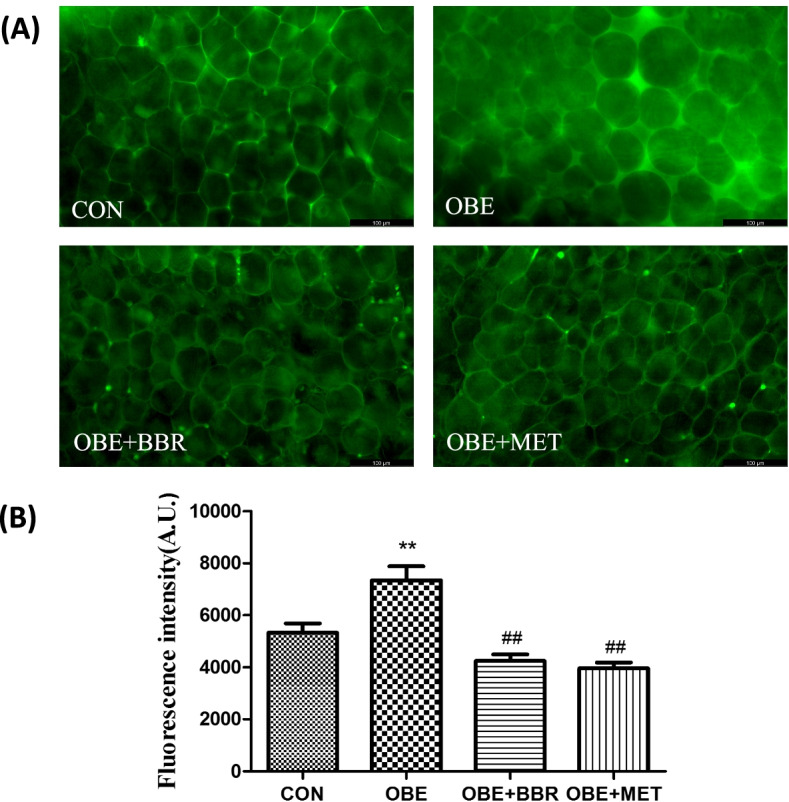
NO in PVAT. **A** Fluorescence expression of NO in secondary perivascular adipose tissue in the mesentery (bars = 100 μm). **B** Fluorescence intensity of NO in perivascular adipose tissue in the mesentery. Data are expressed as the mean ± S.E.M. (*n = *5). ^**^P < 0.01 vs. control rats; ^##^*P* < 0.01 vs. obese rats

The secondary mesenteric blood vessel is tightly connected with PVAT, and it appears in the form of a combined column, including arterioles, venules and the PVAT that surrounds them. We used the whole vascular column for the EFS experiment. Compared with the control rats, the NO release was significantly increased in the obese rats; the release of NA was significantly reduced. All these changes were reversed by BBR and MET (Fig. 5A, B). NO might be derived from the endothelium of the mesenteric vasculature or from the PVAT or nitrergic nerve in PVAT. However, in our experiment, there was no significant change in NO release before and after EFS, indicating that EFS induced little or no neurogenic NO release. This finding also indicates that NA is released from PVAT. Then, we used a fluorescent probe to measure the amount of NO in different groups of PVAT, and the results showed that PVAT is the main source of NO. The fluorescence intensity and the volume of adipocytes in the PVAT in obese rats were significantly increased, and BBR and MET administration reversed this increase (Fig. 6A, B).

### Changes in NOS expression in PVAT

**Fig. 7:**
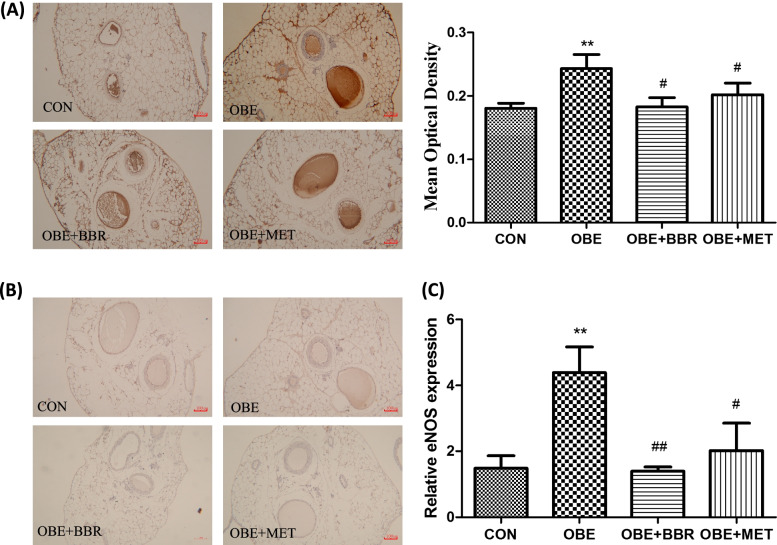
eNOS and iNOS expression. **A** Expression of eNOS proteins in secondary mesenteric vessels (bars = 100 μm). **B** Expression of iNOS proteins in secondary mesenteric vessels (bars = 100 μm). **C** Expression of eNOS mRNA in PVAT. Data are expressed as the mean ± S.E.M. (A, *n = *5–6; C, *n = *3). ^**^P < 0.01 vs. control rats; ^#^*P* < 0.05, ^##^*P* < 0.01 vs. obese rats

Combining network pharmacology analysis and existing experimental results, we observed NOS expression in PVAT. The expression of iNOS proteins was much lower in the secondary mesenteric vessels with PVAT, and there was no significant difference among the groups (Fig. 7A). The expression of eNOS proteins was higher in PVAT, and eNOS was dramatically increased in the obese rats. Furthermore, BBR and MET administration significantly corrected the increase in eNOS (Fig. 7B). Then, we further studied eNOS expression at the transcriptional level. The results of real-time PCR also showed that eNOS mRNA was upregulated in the PVAT of obese rats, and BBR and MET significantly reversed this increase (Fig. 7C).

## Discussion

There were significant changes in the morphology of mesenteric PVAT but not small vessels. These findings indicate that perimesenteric fatty lesions may occur earlier than small arteries in obese rats. Masson staining results of mesenteric slices showed that collagen fibres were increased in obese rats; BBR and MET reversed the changes of adipose tissue and collagen fibres with early intervention. It has been reported that collagen fibres not only played a supporting role in structure, but also were related to the material transport in function [25]. In obese, adipose tissue fibrosis has become an important marker of metabolic disorder in white adipose tissue, and that can damage the plasticity of adipocytes [26, 27]. Luo et al. reported that MET inhibited the excessive deposition of extracellular matrix in white adipose tissue of ob/ob mice and diet induced obese mice; after treated with MET, collagen deposition around adipocytes was reduced; white adipose tissue fibrosis was inhibited, and then improved insulin resistance [28].

The AFM results indicated that vasodilation was increased in obese rats, suggesting changes in systolic and diastolic functions in mesenteric small arteries. Moreover, both BBR and MET reversed this overvasodilation. Our previous studies using the same obesity model showed that there was no significant difference in blood pressure and heart rate between obese and normal rats [29]. Under the same pulse pressure difference, the change in the small vascular diameter becomes the main condition used to determine blood flow. NO also acts as a vasodilator to regulate blood vessel function [30]. The results of network pharmacology showed that eNOS and iNOS are two targets that are highly correlated with obesity and BBR, which supports further exploration of the changes in NO in PVAT.

The organ bath results showed that in mesenteric vasculature with PVAT, NO release was increased, and NA release was significantly decreased in obese rats. However, BBR and MET reversed these changes in NO and NA release. For the assay of NO release, NO might be derived from the endothelium of the mesenteric vasculature, PVAT or the nitrergic nerve in PVAT. However, in our experiment, there was no significant change in NO release before and after EFS, indicating that EFS induced little or no neurogenic NO release. We used a fluorescent probe to measure the amount of NO in different groups of PVAT, and the results showed that PVAT was the main source of NO. Additionally, Gil-Ortega et al. [7] reported that the NO release from mesenteric vascular endothelial cells showed no significant changes in rats with high-fat diet-induced obesity compared with that in control rats. However, the production of NO in PVAT was increased. Reza et al. [31] studied subcutaneous small arteries and found that PVAT modulates vascular contractile tone by releasing vasodilatory mediators, including NO and adiponectin, to have an “anticontractile” effect. In our experiment, the expression of iNOS in blood vessels and PVAT was low; however, the expression of eNOS was much higher. Furthermore, eNOS expression was significantly increased in obese rats. Our results are supported by results found in obese patients. It has been reported [32] that eNOS expression in adipose tissue was ten times greater than that of iNOS, and the PVAT of the mesentery can release NO, which is mainly related to eNOS. There was a report [1] that PVAT was responsible for the increased production of NO by a direct mechanism, and the eNOS isoform was also present in PVAT. NO was directly produced and released, affecting vasculature. NO, as a kind of ROS, is closely related to chronic inflammation in obesity. When NO production is excess, a large number of free radicals, such as reactive nitrogen or ROS, are produced, which can cause tissue damage. In addition, NO also promotes glucose uptake in white adipose tissue [33]. In BBR- and MET-treated rats, the expression of eNOS in PVAT was significantly decreased. Therefore, we think that BBR and MET reduce the expression of eNOS in PVAT, reduce NO production, and improve vascular function. A previous study indicated that BBR restores diabetic endothelial dysfunction through enhanced NO bioavailability by upregulating eNOS expression and downregulating NADPH oxidase expression [34]. However, there have been few reports on the effects of BBR on eNOS expression in obesity models. The mechanism leading to the change in NA needs to be further studied. Many studies support the sympathetic innervation of white adipose tissue (WAT). Bartness and Bamshad [35] proposed that sympathetic nerves may mainly regulate fat mobilization in white adipose tissue. They also proposed that sympathetic nerve stimulation can promote browning of white fat and promote its thermogenesis and fat decomposition. Furthermore, denervation produced significant increases in WAT mass and fat cell number [36]. This may support our results that indicate that NA was decreased in the mesenteric PVAT of obese rats because mesenteric PVAT was thought to be WAT. Ayala-Lopez et al. [37] found that PVAT components that are independent of sympathetic nerves can release NA in a tyramine-sensitive manner, resulting in arterial contraction. This might also explain why there was no change in NA release before and after EFS in our experiment because EFS usually stimulates vascular peripheral nerve fibres, inducing the release of NA.

It is worth noting that in our experiments, the two major factors affecting vascular function changed: NO release increased, and NA release decreased. Therefore, how the two factors influence each other needs further in-depth research. NO is an oxide, and its overproduction will cause tissue damage [38, 39]. We speculate that the increase in NO in adipose tissue leads to damage to sympathetic nerve fibres in PVAT and a decrease in NA synthesis. However, the decrease in NA leads to a decrease in adipose tissue consumption, the promotion of visceral fat accumulation, and the aggravation of chronic inflammatory reactions. BBR and MET can reduce the expression of eNOS and the production of NO. It can then repair the tissue damage caused by excessive NO, improve the synthesis of NA in nerve fibres, and restore the function of adrenergic nerves. The role of NO and ROS (and their potential interaction) in the pathological role of PVAT still require further investigation since antioxidant approaches as therapeutic options have failed to improve PVAT actions in cardiovascular diseases [40].

BBR has played a role in protecting small blood vessels by regulating PVAT. And small blood vessels are spreading throughout the body; its lesions have not received enough attention like large blood vessels and capillaries. In addition, the function of the mesentery directly affects the intestinal tract, and the changes in intestinal function are essential for many diseases such as obesity and diabetes. Therefore, this study has added another confirmation to the multi—target regulation effects of BBR in the aspect of metabolic diseases.

## Conclusions

In summary, we report the novel discovery that the mesenteric small arterioles are over dilated, NO is overproduced, and NA production is decreased in mesenteric PVAT in the early stages of diet-induced obesity in rats. This is related to changes in eNOS in PVAT. All these changes were reversed by BBR and MET, suggesting that BBR ameliorates vascular dysfunction by regulating the overproduction of NO in PVAT. We propose that the study of PVAT may provide a new target for the treatment of vascular dysfunction in diet-induced obesity.

## Data Availability

All data generated or analysed during this study are included in the article.

## Abbreviations

PVAT: Perivascular adipose tissue
HFD: High-fat diet
AFM: Atomic force microscopy
BBR: Berberine
MET: Metformin
EFS: Electrical field stimulation
eNOS: Endothelial nitric oxide synthase
NA: Noradrenaline
NO: Nitric oxide
NOS: Nitric oxide synthase
STZ: Streptozotocin
